# Using cited references to improve the retrieval of related biomedical documents

**DOI:** 10.1186/1471-2105-14-113

**Published:** 2013-03-27

**Authors:** Francisco M Ortuño, Ignacio Rojas, Miguel A Andrade-Navarro, Jean-Fred Fontaine

**Affiliations:** 1 Computer Architecture and Computer Technology Department, University of Granada, C/ Periodista Daniel Saucedo Aranda S/N, Granada, 18071, Spain; 2 Computational Biology and Data Mining, Max Delbrück Center for Molecular Medicine, Robert-Rössle-Str. 10, Berlin, 13125, Germany

**Keywords:** Information retrieval, Text categorization, Citations, Full-text documents, Biomedical literature, Query expansion, Document classification

## Abstract

**Background:**

A popular query from scientists reading a biomedical abstract is to search for topic-related documents in bibliographic databases. Such a query is challenging because the amount of information attached to a single abstract is little, whereas classification-based retrieval algorithms are optimally trained with large sets of relevant documents. As a solution to this problem, we propose a query expansion method that extends the information related to a manuscript using its cited references.

**Results:**

Data on cited references and text sections in 249,108 full-text biomedical articles was extracted from the Open Access subset of the PubMed Central® database (PMC-OA). Of the five standard sections of a scientific article, the Introduction and Discussion sections contained most of the citations (mean = 10.2 and 9.9 citations, respectively). A large proportion of articles (98.4%) and their cited references (79.5%) were indexed in the PubMed® database.

Using the MedlineRanker abstract classification tool, cited references allowed accurate retrieval of the citing document in a test set of 10,000 documents and also of documents related to six biomedical topics defined by particular MeSH® terms from the entire PMC-OA (p-value<0.01).

Classification performance was sensitive to the topic and also to the text sections from which the references were selected. Classifiers trained on the baseline (i.e., only text from the query document and not from the references) were outperformed in almost all the cases. Best performance was often obtained when using all cited references, though using the references from Introduction and Discussion sections led to similarly good results. This query expansion method performed significantly better than pseudo relevance feedback in 4 out of 6 topics.

**Conclusions:**

The retrieval of documents related to a single document can be significantly improved by using the references cited by this document (p-value<0.01). Using references from Introduction and Discussion performs almost as well as using all references, which might be useful for methods that require reduced datasets due to computational limitations. Cited references from particular sections might not be appropriate for all topics. Our method could be a better alternative to pseudo relevance feedback though it is limited by full text availability.

## Background

Retrieving information from the biomedical literature involves the identification and analysis of documents from millions indexed in public databases such as PubMed [1]. The size of this widely used database has a negative impact on the relevance of users’ query results; simple free-text queries would return many false positives. Additionally, when reading a document of interest, users can query for related documents. Query expansion or reformulation is used to improve retrieval of documents relevant to a free-text query or related to a document of interest.

Various query expansion or reformulation strategies have been proposed in the biomedical or genomics field [2-5]. A user’s free-text query defining the need for some information can be enriched with common synonyms or morphological variants from existing or automatically generated thesauruses, terms can be weighted, and can also be corrected for spelling errors. By default in PubMed, free-text queries are reformulated with Medical Subject Headings (MeSH) terms. The MeSH thesaurus is a biomedical controlled vocabulary used for manual indexing and searching PubMed. Relevance feedback methods involve the user in selecting relevant documents from results of an initial query in order to reformulate it, and pseudo relevance feedback (PRF) methods consider the top documents returned by the initial query as relevant in order to reformulate the query, avoiding additional user interaction [6].

Alternatively, content similarity algorithms are used to compare biomedical documents. When applied on freely available abstracts in PubMed, such algorithms use words, as well as other features available in indexed abstracts (e.g. authors list, journal title, and MeSH terms) or features processed by specific algorithms (e.g. part of speech, semantic processing) [7-12]. However, when a single document is used as input (as for the PubMed Related Articles (PMRA) algorithm used to display a list of related documents in PubMed [13]), its abstract might not have enough content to allow proper retrieval. Using the full text offers one possibility for expanding the information related to one document, and is increasingly used as more full text manuscripts become available from large resources such as the PubMed Central (PMC) database and its Open Access subset (PMC-OA) [4,14]. Another possibility is given by the references associated to the article by citation: either cited documents or documents citing it. For a given scientific document, finding the cited references is straightforward since they are usually listed in a dedicated section. In contrast, finding its referring citations requires mining all existing scientific documents, which might be impractical.

Using related references by citation has been already used for classification of documents. For example, it was shown that algorithms based on shared references or citations can outperform text-based algorithms in a digital library of computer science papers [15]. Papers were compared using three bibliometric similarity measures: co-citation (based on the number of citing documents in common) [16], bibliographic coupling (based on the number of cited documents in common) [17], or both [18]. Similarly, it was shown that citation-based algorithms performed better than non-citation-based algorithms such as PMRA in a small dataset of surgical oncology articles [19]. Ranking algorithms were based on impact factors, citation counts and Google™’s PageRank [20]. However, the opposite conclusion was drawn in another document clustering task [21], i.e. citation-based algorithms performed worse than text-based algorithms. Authors used a graph-based clustering technique that groups documents with respect to their connections to other documents in the citation graph. Sentence level co-citations were also shown to be relevant for finding related articles [22]. Articles were related to each other by graph random walks in a co-citation graph. Also, the citation context (words surrounding a citation in a text paragraph) provides different information than the cited abstract [23] and was used for classification [21-24].

References in scientific documents may contain relevant and related information but their usefulness in retrieving related documents (or in classifying related versus non-related documents) from large sets of biomedical documents, starting from a query formed by one single manuscript, still has to be demonstrated.

In this article, we have studied articles in PMC-OA and the impact of using the text from their referenced documents by a query expansion method. We tested different subsets of references and observed that cited references indeed improve the task of retrieving documents related to a single document.

## Methods

### PubMed abstracts

PubMed citations were downloaded in XML format and data was extracted only from citations with an abstract in English. The extracted data relevant to the present study was composed by the PubMed Identifier (PMID), the title, the abstract, and the MeSH annotations. The latter were extracted from XML tag DescriptorName having option MajorTopicYN value equal indifferently to ‘Y’ or ‘N’. A list of nouns from both the title and the abstract was generated by the TreeTagger part-of-speech processor (tags "NN", "NR", "NNS", "NRS", "NP", or "NNPS") [25]. A stop word list was used to filter out common and irrelevant terms. These lists of nouns were used as classification features by the MedlineRanker algorithm (see details below).

### PubMed central open access subset (PMC-OA) full-text documents

Information related to references (cited documents) in a document was extracted from the Open Access subset of PubMed Central (PMC-OA) [1], a biomedical literature database of full-text documents. They were downloaded in XML format (date: 14 September 2011) and parsed to extract the following data stored in a local MySQL (v5.1.49) database: title, PMID, authors, date, document section, and type of document. After removing overlapping and not well formatted XML documents where standard tags cannot be identified, 249,108 documents were retained for analysis. Document sections were identified by keywords that appear in their header (Table 1). The most common sections were represented within the following classification: ‘Introduction’, ‘Materials and Methods’, ‘Results’, ‘Discussion’ and ‘Conclusions’. Headers that could not be assigned to any of these sections or that could be assigned to several (e.g. ‘Results and Discussion’) were labelled as ‘Unknown’.

**Table 1:** Keywords used to identify standard sections

**Standard sections**	**Keywords**
Introduction	Introduction, Background, Review, Context, Literature
Materials and Methods	Methods, Materials, Implementation, Experimental
Results	Results, Findings
Discussion	Discussion
Conclusion	Conclusion(s)

### Document classification

Document classification was performed by the MedlineRanker web tool [26], which processes biomedical abstracts from PubMed. MedlineRanker implements a linear naïve Bayesian classifier that is trained on a set of documents representing a topic of interest (the training set) in comparison to random documents or the rest of PubMed (the background set). After training, the algorithm ranks a third set of documents (the test set). Each set is defined as a list of relevant PubMed identifiers (PMIDs). Nouns in abstracts are used as classification features (using in addition verbs or adjectives was shown to be detrimental to classification performance [11]). Full text, annotations or metadata (e.g. MeSH terms, authors or journal data) are not taken into account. Counting multiple instances of nouns in the same abstract was shown not to improve performance significantly [27] and is not used by MedlineRanker. For each scored abstract, an associated p-value is defined as the proportion of documents with higher scores within 10,000 random recent abstracts.

We used a local database to build queries to the MedlineRanker SOAP web service (Release 2012-07-01; http://cbdm.mdc-berlin.de/tools/medlineranker/soap/). Our database provided the training set as PMIDs of documents cited in a query document. Background sets were composed of random articles or the rest of PubMed. In all the benchmarks, we used non overlapping training and background sets, and test sets were processed using a leave-one-out cross validation procedure. Scripts and statistical analyses of the data mining method were programmed using Perl 5.10.1 and R 2.13.1 [28]. It is important to note that MedlineRanker processes only PubMed abstracts, and that information on cited documents was used only to build appropriate training sets.

### Benchmark 1

A first benchmark was performed to assess if references, used to train a classifier, allow accurate classification of the citing document from a large set of random documents. A total of 10,000 articles were randomly selected for this test. For each of them, we built a training set composed by PMIDs of its references, which was used to rank the article with respect to the rest of PubMed. As mentioned above, we prepared the training, background and test datasets so that they had no single document in common.

### Benchmark 2

In a second benchmark, we assessed the usefulness of references to retrieve documents related to the topic described in the citing document. Manual annotations of PubMed entries with MeSH terms provide accurate sets of topic-related documents (used as gold standards here for document classification). We selected six topics represented by the following MeSH terms: 'Breast Neoplasms', 'Alzheimer Disease', 'Stem Cells', 'Phosphorylation', 'Oligonucleotide Array Sequence Analysis' and 'Randomized Controlled Trials as Topic'. There were 3426, 602, 1093, 3007, 5834 and 1317 PMC-OA articles annotated with these MeSH terms, respectively. The task consisted in finding related documents to a query document by classifying the PMC-OA dataset in two sets of related and non-related documents.

For each MeSH term M, we built a list of positive PMC-OA documents (annotated with M), a list of negative PMC-OA documents (not annotated with M), and a background set (50,000 PubMed abstracts not annotated with M and not in PMC).

For each positive document, we built several training sets composed by either its own PMID, PMIDs of all of its references, or PMIDs of references cited in particular sections. Then, MedlineRanker was trained with each training set and the background set to rank the all PMC-OA (positive and negative documents). The overlap between different sets, including cited articles in the training set, was removed before training. Results obtained using only the abstract of the query document and not its references were taken as baseline.

Given a p-value threshold, MedlineRanker returns a list of candidate abstracts from the test set. That list of candidates includes a set of true positives if they belong to the positive set and a set of false positives otherwise. The true positive rate (i.e. the sensitivity) is defined as the number of true positives in the list of candidates divided by the total number of positives. The false positive rate is measured as the number of false positives in the list of candidates divided by the total number of negatives. Classification performance is then measured by the area under the receiver operating characteristic (ROC) curve. Mann–Whitney U tests [29] were used to compare distributions of areas under the ROC curve. Tests having a p-value below 0.01 were considered significant.

### Comparison to pseudo relevance feedback (PRF)

In Benchmark 2, we also compared our proposed query expansion method using all cited references of the query document to PRF. As described above, for each MeSH term M, a positive, a negative and a background set were defined. For each positive document, the query expansion was defined by the top 20 PMC-OA documents returned by an initial ranking of all PMC-OA documents using the single positive document for training versus the background set. The positive document and the additional 20 PMC-OA documents were then used to train MedlineRanker versus the background set to rank the all PMC-OA. Only this second ranking was evaluated by the area under the ROC curve.

### Scoring schemes

MedlineRanker uses a naïve linear Bayesian classifier. For comparison, we have implemented PMRA and BM25 formulas in MedlineRanker. Formulas of PMRA and BM25 apply to the comparison of only two documents. In MedlineRanker, each document from the test set is compared to the training set which could contain several documents. In this case, documents of the training set are merged and considered as a single one.

#### MedlineRanker

The MedlineRanker algorithm consists on comparing noun usage into a set r of relevant abstracts (from the training set) of size N_r_ and a set r’ of irrelevant abstracts (from the background set) of size N_r’_ (see [10,26] for more details). For a given abstract, each feature i (here nouns) is given a weight W_i_ by the following formula:

$$W_{i}=\frac{T_{r,i}}{1-T_{r,i}}/\frac{T_{r',i}}{1-T_{r',i}}$$

It is the refactored-for-speed weight which allows summing of only nouns that occur in the abstract [30], where the posterior estimate of the frequency of feature i in relevant documents T_r,i_ is defined as:

$$T_{r,i}=\frac{N_{r,i}+2T_{r}}{N_{r}+4T_{r}}$$

This estimate uses the split-Laplace smoothing introduced in [31] to counteract class skew, where N_r,i_ is the occurrence of noun i in relevant documents, and the Laplace-smoothed probability of relevance T_r_ is defined as:

$$T_{r}=\frac{N_{r}+1}{N+2}$$

where N is the total number of documents. T_r',i_, and T_r’_ are obtained from the same formulas by replacing r by r’ where N_r’,i_ is the occurrence of noun i in irrelevant documents.

Finally, the score of a given abstract A is the sum of its noun weights:

$$\text{Score}\text{MedlineRanker}=\underset{i\in A}{\sum }W_{i}$$

#### PubMed related articles (PMRA)

We implemented the scoring function PMRA with optimal parameters from [13], defining the similarity of document c and d:

$$\text{Score}\mathrm{PMRA}=\underset{t\in c}{\sum }w_{t,c}*w_{t,d}$$

where w_t,c_ is the weight of term t in document c. This weight is defined as:

$$w_{t}={\left(1+{\left(\frac{\mu }{\lambda }\right)}^{k-1}e^{-\left(\mu -\lambda \right)l}\right)}^{-1}\sqrt{\mathrm{id}f_{t}}$$

where μ=0.022 and λ=0.013 as proposed in [13], k is the number of occurrences of term t in the document, l is the length of the document in words, and idf_t_ is the inverse document frequency for term t defined as:

$$\mathrm{id}f_{t}=\log\left(\frac{1+\text{number}\;\text{of}\;\text{documents}}{1+\text{number}\;\text{of}\;\text{documents}\;\text{containing}\;\text{term}\;t\;}\right)$$

#### Okapi BM25

We implemented the scoring function called Okapi BM25 [32,33] based on the formula used in [34]. This score comparing document q and d is defined as:

$$\mathrm{Score}\;\mathrm{BM}25\left(q,d\right)=\underset{i\in d}{\sum }\left({\mathrm{IDF}}_{i}*\frac{n_{i}\left(k_{1}+1\right)}{n_{i}+k_{1}\left(1-b+b\frac{\left|D\right|}{\mathrm{avg}\left(\left|D\right|\right)}\right)}\right)$$

where the n_i_ is the frequency of term i in document d. *|D|* is the length of the document d in words, avg(|D|) is the average document length, and *b*=1.0 and *k*_*1*_=1.9 as proposed in [13]. IDF_i_ is the inverse document frequency of term i defined as:

$$\mathrm{ID}F_{i}=\log\left(\frac{0.5+N-d_{i}}{0.5+d_{i}}\right)$$

where N is the total number of documents in the dataset and d_i_ is the number of documents containing term i.

## Results

As training an accurate classifier with only one document is a challenging task and reflects real use cases, we have tested the relevance of using freely accessible data from referenced documents (scientific abstracts). After analyzing data on documents and references from the PubMed Central Open Access subset (PMC-OA), we have addressed the following questions: are references cited in a document relevant to discriminate this document (or related documents) from random ones? Is the relevance of cited references to classify the citing document dependent on the section in which they appear? We have also compared this method with pseudo relevant feedback and several scoring schemes.

### PubMed Central open access subset data

A local database was first built to store data of 249,108 open access documents from PMC-OA. For each document, information about its cited references, including in which manuscript section they were cited, was also included in the database. A total of 13,737,573 references to cited documents were then retrieved (this count includes multiple citations to the same reference). Finally, we stored in the database the list of topics defined by MeSH annotation (from PubMed) associated to each article.

Of all PMC-OA documents, 98.4% were covered by PubMed (e.g. had an annotated PMID). The most common document types were research article (202,520 occurrences), review article (17,962 occurrences), and case report (8,854 occurrences) (Table 2). They were largely covered by PubMed (99.5%, 98.9%, and 98.5% respectively).

**Table 2:** Types of documents from the PMC open access subset

**Document type**	**# Documents**	**PubMed coverage (%)**
research-article	202520	99.5
review-article	17962	98.9
case-report	8854	98.5
letter	3268	90.5
Editorial	2921	95.3
other	13583	85.2
TOTAL	249108	98.4

Of all references cited in PMC-OA documents, 79.5% were covered by PubMed. The most common reference types were journal (12,415,337 occurrences), book (649,775 occurrences), and web page (21,523 occurrences). Only references to journal documents were largely covered by PubMed (87.8%, 1.1%, and 0.1% respectively; see Table 3).

**Table 3:** Types of cited references from the PMC open access subset

**Reference type**	**# References**	**PubMed coverage (%)**
journal	12415337	87.8
book	649775	1.1
webpage	21523	0.1
conference proceedings	15499	1.1
web	8711	2.7
others	626728	3.5
TOTAL	13737573	79.5

**Figure 1:**
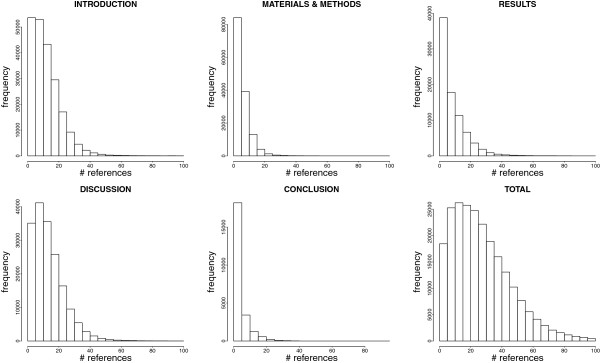
**Cited references distributions.** Data from the open access subset of PubMed Central shows that the distribution of cited references varies between full documents (Total) and across sections. The y-axis shows the number of PMC-OA documents.

In benchmarks shown below, training sets can be composed of articles cited from different sections. In principle, the more documents in the training set, the better the classification. Therefore, we examined in more details the distributions of references per section (Figure 1). Full text documents contained on average 27.2 references. ‘Introduction’ and ‘Discussion’ sections contained a fair average number of references (10.2 and 9.9, respectively). Fewer references were obtained from the ‘Conclusion’, ‘Results’ or ‘Materials and Methods’ sections were fewer (maximum = 3.4).

### Benchmark 1: retrieving a document using its references

**Figure 2:**
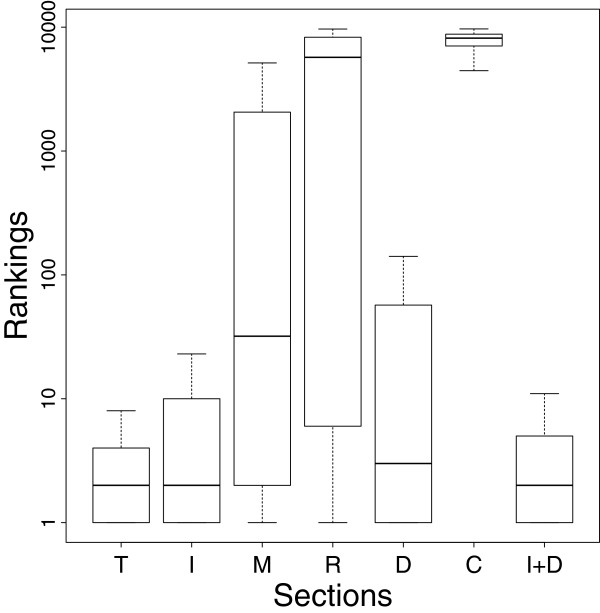
**Classification of the citing document using cited references.** The MedlineRanker algorithm was used to rank the citing document with respect to random ones using the references cited in the full document or its sections. Ranks were calculated by MedlineRanker for each document of a test set of 10,000 documents. T: total (or full document), I: introduction, M: methods, R: results, D: discussion, C: conclusion, and I+D: introduction and discussion.

The first benchmark was performed to determine if references cited by a document allow the classification of the citing document from a set of random documents (see Methods for details). In principle, the set of references or a subset of it is expected to be strongly associated with the same topics of the original document. For this benchmark we used a test set composed of 10,000 randomly chosen documents. MedlineRanker was used to rank each document with respect to the whole set of 10,000 documents using the references cited in various sections. The output ranks of these documents were analyzed (Figure 2).

Using references from the full text (T) provided the best rankings for the citing documents followed by ‘Introduction and Discussion’ (I+D), ‘Introduction’ (I) (third quartile lesser or equal to rank 10). Using the ‘Discussion’ (D) led to more variability, though the median rank was still below 10. Other sections showed clearly worse results, with the ‘Methods’ and ‘Results’ sections showing very high variability, and the ‘Conclusion’ being totally irrelevant. These results show that references are highly related to the topic of the article where they are cited. Therefore, they could be used to retrieve more documents related to the citing document.

### Benchmark 2: retrieving topics-related documents using references from a single document

Next, we wanted to evaluate how the performance of topic-related document retrieval from a single query document supplemented with cited references is affected by the topic of the query document. We chose six particular topics in PMC-OA documents represented by their MeSH annotations (See Methods for details). Each PMC-OA document related to these topics (i.e. annotated with a selected MeSH term) and its cited references were used to classify the rest of PMC-OA in two sets of topic-related and non-topic-related documents.

**Figure 3:**
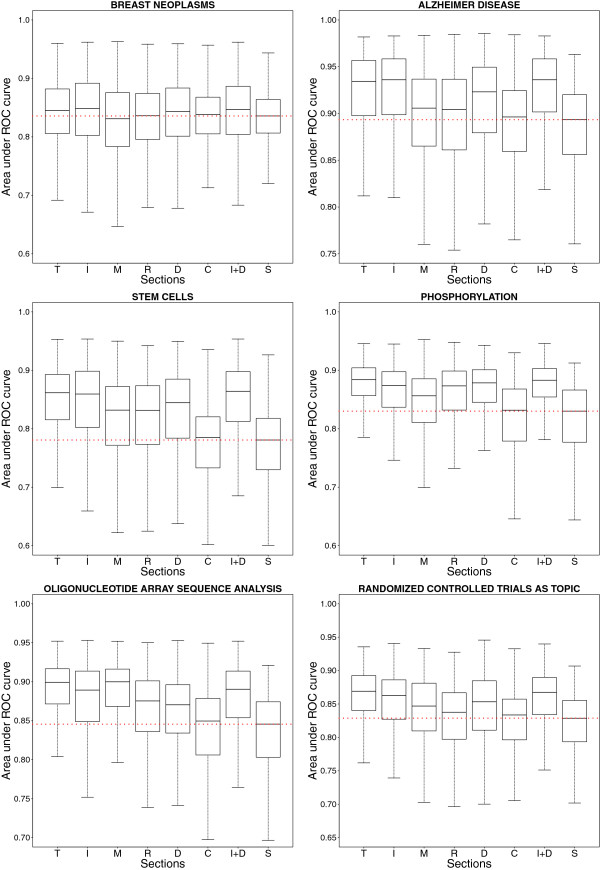
**Classification of topics-related documents.** In this classification task, the algorithm ranked documents with a given MeSH term annotation with respect to random documents using the references cited in the document and present in the full document or its sections. The median ROC area obtained when training only with the document of interest (S, the baseline) is indicated (dotted line). T: total (or full document), I: introduction, M: methods, R: results, D: discussion, C: conclusion, and I+D: introduction and discussion, ROC: receiver operating characteristic.

Comparing the distributions of ROC areas, training sets composed by cited references from the full text (T), the ‘Introduction’ (I) or the ‘Discussion’ (D) always returned significantly better results (p-value<0.01, one-sided Mann–Whitney U test) than the baseline (S) (Figure 3), with higher effect size than other training sets except for topic ‘Oligonucleotide Array Sequence Analysis’ where references from the ‘Methods’ and ‘Results’ sections performed well.

For this reason, references from the ‘Introduction’ or the ‘Discussion’ sections were also joined in an additional training set (‘Introduction and Discussion’), which performed similarly to using the set of all references.

The ‘Conclusion’ training set showed non significant results (p-value>0.01, one-sided Mann–Whitney U test) except for topics ‘Breast Neoplasms’ and ‘Oligonucleotide Array Sequence Analysis’ although medians were very close to the baseline (fold change equal to 1.004 and 1.006 respectively). This could be expected from the low number of references associated to this section (See Figure 1).

Note that performances reported above for references taken from each section correlate with the number of documents sharing the query MeSH annotation (Table 4). Interestingly, for the term ‘Randomized Controlled Trials as Topic’, we found very few cited documents sharing the annotation but classification performances were still good. This highlights the usefulness of algorithms that do not use annotations but only words in text.

**Table 4:** Percentages of cited documents sharing a MeSH annotation with the citing document

**MeSH term**	**Total**	**Introduction**	**Materials & Methods**	**Results**	**Discussion**	**Conclusion**
Breast Neoplasms	45.35	45.43	39.15	39.01	48.65	53.65
Alzheimer Disease	43.32	48.22	31.39	33.94	46.56	51.76
Stem Cells	38.81	41.74	32.20	36.88	39.13	38.46
Phosphorylation	23.04	20.99	17.46	27.84	24.62	15.83
Oligonucleotide Array Sequence Analysis	19.51	20.67	30.47	15.52	15.09	31.50
Randomized Controlled Trials as Topic	0.15	0.15	0.17	0.05	0.20	0.13

#### Comparisons

**Figure 4:**
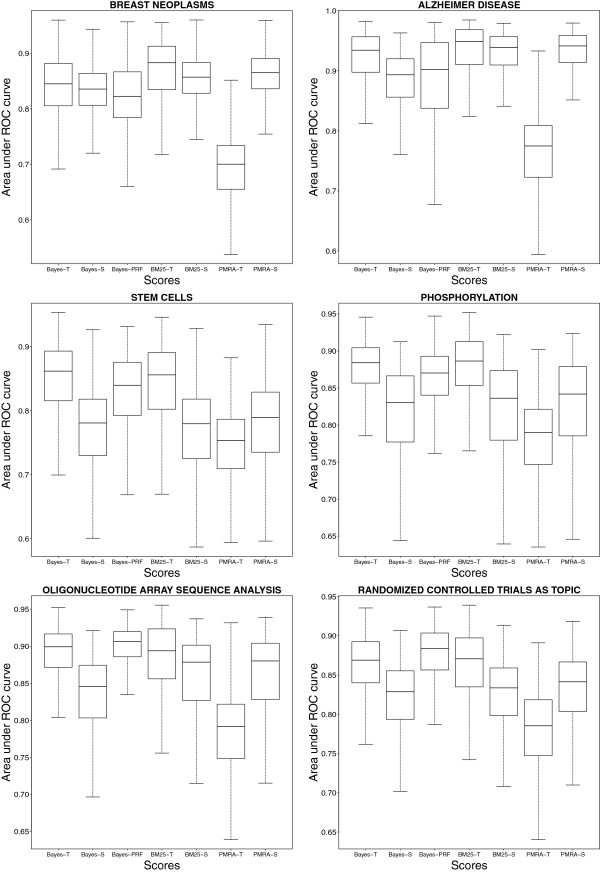
**Comparisons.** The second benchmark was reproduced using two other scoring schemes (Okapi BM25 and PMRA) for comparison with MedlineRanker (Bayes). Results were produced training with only the document of interest (S, the baseline) or training with all references from the full text (T). An alternative query expansion method based on pseudo relevance feedback using the MedlineRanker scoring scheme was also compared (Bayes-PRF). ROC: receiver operating characteristic.

Additionally to the built-in MedlineRanker scoring scheme based on naïve Bayesian statistics, PMRA and Okapi BM25 scoring schemes were also used for comparison in Benchmark 2. We produced results for the baseline (S) and for the training sets composed by all cited references (T) (Figure 4). On the baseline, all scoring schemes showed close results although PMRA’s median was often slightly higher. MedlineRanker and BM25 scoring schemes produced always significantly better results than their respective baselines (p<0.01, two-sided Mann–Whitney U test). On the contrary, results for PMRA were always significantly worse than the baseline (p<0.01, two-sided Mann–Whitney U test).

We compared our proposed query expansion method using cited references to an implementation of PRF. Expansion used text from top 20 returned documents from an initial query based on the single query document. PRF was significantly better than the baseline in 5 topics and significantly worse in one topic (for Breast Neoplasms). Our method significantly outperformed PRF in 4 topics (p<0.01, two-sided Mann–Whitney U test), and PRF was significantly better in 2 topics (Oligonucleotide Array Sequence Analysis, and Randomized Controlled Trials as Topics) but with lower fold changes (1.012 and 1.018, respectively).

## Discussion

A simple and popular request in document retrieval is to find the bibliography related to one single document, as implemented in the PubMed Related Articles (PMRA) feature [13]. Text mining algorithms for document retrieval are optimally trained with large enough sets of relevant text [26], thus using a training set composed of one single article is challenging. Here we have evaluated the potential of the expansion of single article training sets with the bibliography cited in the article. As shown in a previous study about keyword content in full text biomedical articles [35], manuscript sections are relevant in the retrieval of the article topic. Thus, we also explored how retrieval of related documents depends on the use of references from different manuscript sections.

While the PubMed biomedical literature database contains millions of freely available abstracts, information on cited references was found in 249,108 full text documents from the Open Access subset of the PMC database [1]. Consequently, the proposed approach is limited by accessibility to full-text documents. Note that the number of open access PMC articles is currently too small for some text mining studies. For instance, the BioCreative III challenge first intended to run a text mining competition using full text articles to extract or retrieve protein-protein interaction (PPI) data or documents relevant to PPI information but finally only abstracts were used due to the very small overlap between PMC and known manuscripts cited in PPI databases [36].

The size of PMC-OA was also too small to have an interesting overlap with existing text corpora such as from the TREC Genomics Tracks and OHSUMED [2,3,37]. Consequently, we have used MeSH term annotations to define related documents as documents sharing a same MeSH term. This could be refined taking advantage of the MeSH vocabulary hierarchy, including for example children terms. Our second benchmark could be seen as a MeSH indexing task for which various methods were proposed (see for example [38-43]). Differently from these methods, we focused only on selected topics representing exemplary biomedical research fields avoiding general topics such as ‘Human’ or ‘Europe’; we also did not investigate the indexing of several or all MeSH terms simultaneously. Different benchmarks would be needed in order to compare our method to existing MeSH indexing algorithms.

While availability of full text is a limitation, mapping of references to PubMed is not an issue for most PMC-OA documents and their cited references as shown by our study (Table 2 and Table 3). Moreover, the average number of references in documents (27.2) shows clear potential for improving classification results (Figure 1) [10,26].

We demonstrated that cited references found in a document can accurately discriminate this document from a random set (Figure 2). Using all references led to better results than using the baseline (the query document only) or references cited from particular sections. However, it was interesting to note that gathering references from ‘Introduction’ and ‘Discussion’ showed similar performance (Figure 2 and Figure 3): these two sections may contain most of the topic-related data useful for classification [44]. This is supported by the higher number of citations found in these sections (Figure 1) and the enrichment of these cited references in similar MeSH annotations (Table 4). This result may be of interest for users of Support Vector Machines or other similarly computing-intensive methods, since reducing the number of documents or features in the training set would shorten the training procedure without affecting performance [45,46].

Query expansion by citation context was already shown to be effective [21,24] although terms from citation context describe general aspects of the topic of an article and classification performance may decrease with topic specificity [21]. Topic-dependent results were also found in MeSH indexing [47,48]. In our study, we also noted that classification performance using references by section was dependent on the topic. In general, ‘Methods’ and ‘Results’ sections performed worse. But, these sections performed better for the technical topic ‘Oligonucleotide Array Sequence Analysis’ (Figure 3). The decision to limit the use of cited references to a given section to train a text classifier must therefore depend on the topic. The choice of the scoring scheme is also critical since the query expansion could be detrimental to the performance, such as for the PMRA scoring scheme. Notably, the implementation of the latter is based on a publication from 2007 [13] and differs from the current version available to PubMed users (which uses also MeSH terms as classification features).

Comparison to an implementation of pseudo relevance feedback (PRF) was significantly favourable to our method in 4 (66.7%) out of 6 topics. Contrary to our method, PRF was not systematically better than the baseline but other implementations of PRF may perform better, especially when weighting differently text from pseudo relevant documents [49]. Nevertheless, a major advantage of PRF is that it is not limited by access to full-text documents.

While we have focused on the biomedical field, it would be interesting to generalize its conclusions to other fields; this would need further benchmarks. Only PubMed and PMC-OA were used as source of text and references data while other databases may be valuable such as Google Scholar or private content from some scientific publishers. Nevertheless, we have used the largest biomedical resources providing free content and widely used by the community, which allow reproducing and studying ways to improve upon our results. Only few selected topics were analyzed in detail, though they represented different biomedical research fields and the first benchmark (Figure 2) could be considered as a topics-independent proof of concept. Still, we have observed some degree of topic-specific behaviour, but a more thorough study including more topics may reveal interesting results.

## Conclusions

In conclusion, we have demonstrated the usefulness of cited references to expand text used by classifiers using as input a single document. Choosing all cited references is the safest choice while references from a particular section might not be suited for some topics. Implementation of such method may be limited by access to full-text articles or data on cited references, but can significantly outperform pseudo relevance feedback methods (p-value<0.01) and will further improve in the near future due to the growth of the open access scientific literature.

## Abbreviations

PMRA: PubMed Related Articles; PMC: PubMed Central; PMC-OA: PubMed Central Open Access subset; PMIDs: PubMed identifiers; MeSH: Medical Subject Headings; ROC: Receiver Operating Characteristic; PRF: Pseudo Relevance Feedback

## Competing interests

The authors declare that they have no competing interests.

## Authors’ contributions

Conceived and designed the experiments: J-FF, FMO and MAA-N. Performed the experiments: J-FF and FMO. Analyzed the data: J-FF, FMO and MAA-N. Wrote the manuscript: J-FF, FMO, IR and MAA-N. All authors read and approved the final manuscript.
